# Watershed infarction during the first favism crisis in a Syrian child; a rare case report

**DOI:** 10.1186/s12887-026-07550-7

**Published:** 2026-08-19

**Authors:** Sanaa Alakari, Nawar Mansour, Enas Akash, Ahlam Khosrouf, Bassam Nader, Mohammad Samih Khallouf

**Affiliations:** 1 Department of Pediatrics, Homs University Hospital, Homs, Syrian Arab Republic; 2 Department of General Surgery, Homs University Hospital, Homs, Syrian Arab Republic; 3 Department of Internal Medicine, Homs University, Homs, Syrian Arab Republic

**Keywords:** Watershed infarction, G6PD deficiency, Hemolytic crises, Favism, Case report

## Abstract

**Background:**

A watershed infarction is an ischemic event within the border zone regions of the brain. It can be associated with hematologic disorders including beta thalassemia and sickle cell disease. However, it has not been reported during a favism crisis.

G6PD deficiency is an X-linked recessive genetic disorder and one of the most prevalent enzyme deficiencies in humans, with a global prevalence of 4.9%. The most common triggers for hemolytic crises in G6PD deficiency are fava bean ingestion, infection and specific medications. We describe a rare case of a 3-year-old boy who developed a watershed infarction during his first favism crisis — an association has not previously reported.

**Case presentation:**

A 3-year-old male was admitted to our hospital with an acute hemolytic crisis (with a hemoglobin level of 3.3 g/dL) occurred after fava bean ingestion. After 6 hours of admission the patient developed neurological symptoms with normal vital signs and no hypotension. After several investigations, bilateral watershed infarction was confirmed by Magnetic Resonance Imaging. Fluid resuscitation and blood transfusion with intensive care were sufficient to improve the neurological symptoms and elevate hemoglobin level above 10 g/dL. The patient was discharged after 9 days of admission with full recovery.

**Conclusion:**

According to our acknowledgement, this is the first reported case of watershed infarction concurrent with favism, thus we aim to highlight the importance of accurate and frequent neurological examination for hemolytic crises patients to prevent adverse complications of delayed treatment.

**Supplementary Information:**

The online version contains supplementary material available at https://doi.org/10.1186/s12887-026-07550-7.

## Background

A watershed infarct refers to an ischemic event occurring within the border zone regions [1], and is characterized by distinctive features on brain imaging [2]. Etiological factors for watershed infarction include: cardiac decompensation, vasculopathies, blood pressure dysregulation (both hypotension and hypertension), dyslipidemia, carotid stenosis, and hematologic disorders [2]. Watershed infarction in patients with β-thalassemia [1], and sickle cell disease [3] represent a rarely documented clinical entity. A causal association between this type of infarction and episodes of hypotension has been confirmed [2]. 

Glucose-6-phosphate dehydrogenase (G6PD) deficiency is recognized as one of the most prevalent human enzyme deficiency disorders worldwide. Current epidemiological data indicate an overall global prevalence of approximately 4.9% [4]. It is an X-linked recessive genetic disorder; therefore, hemizygous males are more susceptible to severe clinical complications [5]. 

The primary triggers for hemolysis include a specific set of contraindicated medications, in addition to infections and the ingestion of fava beans, which are among the most common causative agents [5]. 

The onset of symptoms is not invariably concomitant with the initial exposure to fava beans, and the severity of the symptoms can be attributed to the disproportionate ratio between the volume of beans consumed and the patient’s body mass [6]. 

The clinical presentation consistently demonstrates an underlying acute hemolytic anemia, constituting a pathognomonic feature of favism. Laboratory analysis is characterized by evident anemia [6]. 

The diagnosis of G6PD deficiency is confirmed through genetic testing [5]. The therapeutic interventions consist of hydration with normal saline solution and urgent blood transfusion (packed red blood cells RBCs). Prevention is achieved through strict trigger avoidance and the provision of genetic counseling for families [5]. In this article, we present the case of a 3-year-old male who developed a watershed infarction during his first favism crisis, which, to the best of our knowledge, has not been previously reported.

## Case presentation

A 3-year-old male presented to the emergency department with sudden-onset jaundice, pallor, dark urine, fever, and fatigue, which have been started one day prior, following his first consumption of raw fava beans two days earlier. Family history was significant for favism in his mother and maternal uncle.

On examination, he was lethargic but responsive, with marked pallor and jaundice. Vital signs showed tachycardia (HR 160 b/m), tachypnea with hypoxia (oxygen saturation 88% improving with oxygen therapy), and mild hypotension (105/48 mmHg). Cardiac examination revealed a systolic murmur, and the radial pulse was of medium volume bilaterally. Abdominal and neurological exams on admission were unremarkable, as no hepatosplenomegaly was detected.

Laboratory findings confirmed severe hemolytic anemia, with a hemoglobin level of 3.3 g/dL, white blood cells 29,460/mm^3^, neutrophils 76%, MCV 89 fl., PLT 229 × 10^3^/mm^3^, elevated reticulocytes (12%), and a total bilirubin of 7.5 mg/dL (primarily indirect 6 mg/dL). Urinalysis was positive for urobilinogen and showed heavy hemoglobinuria (+++). Blood urea nitrogen was elevated at 82 mg/dL, while serum creatinine, platelet count, blood glucose, prothrombin time (PT), and partial thromboplastin time (PTT) were within normal limits. Peripheral blood smear showed anisopoikilocytosis, schistocytes (helmet cells), bite cells, and nucleated RBCs. Blood transfusion was initiated two hours after admission and the patient received a bolus of normal saline and two doses of packed RBCs transfusion of 15 mL/kg per dose.

Six hours post-admission, the patient developed acute neurological deterioration featuring agitation, confusion, non-purposeful involuntary movements, orofacial dyskinesia (lip-smacking), and slowed speech. The Glasgow Coma Score (GCS) was 10/15.

Muscle strength was slightly reduced in the upper limbs, graded 3/5, and was severely weak (0/5) in the lower limbs.

**Fig. 1:**
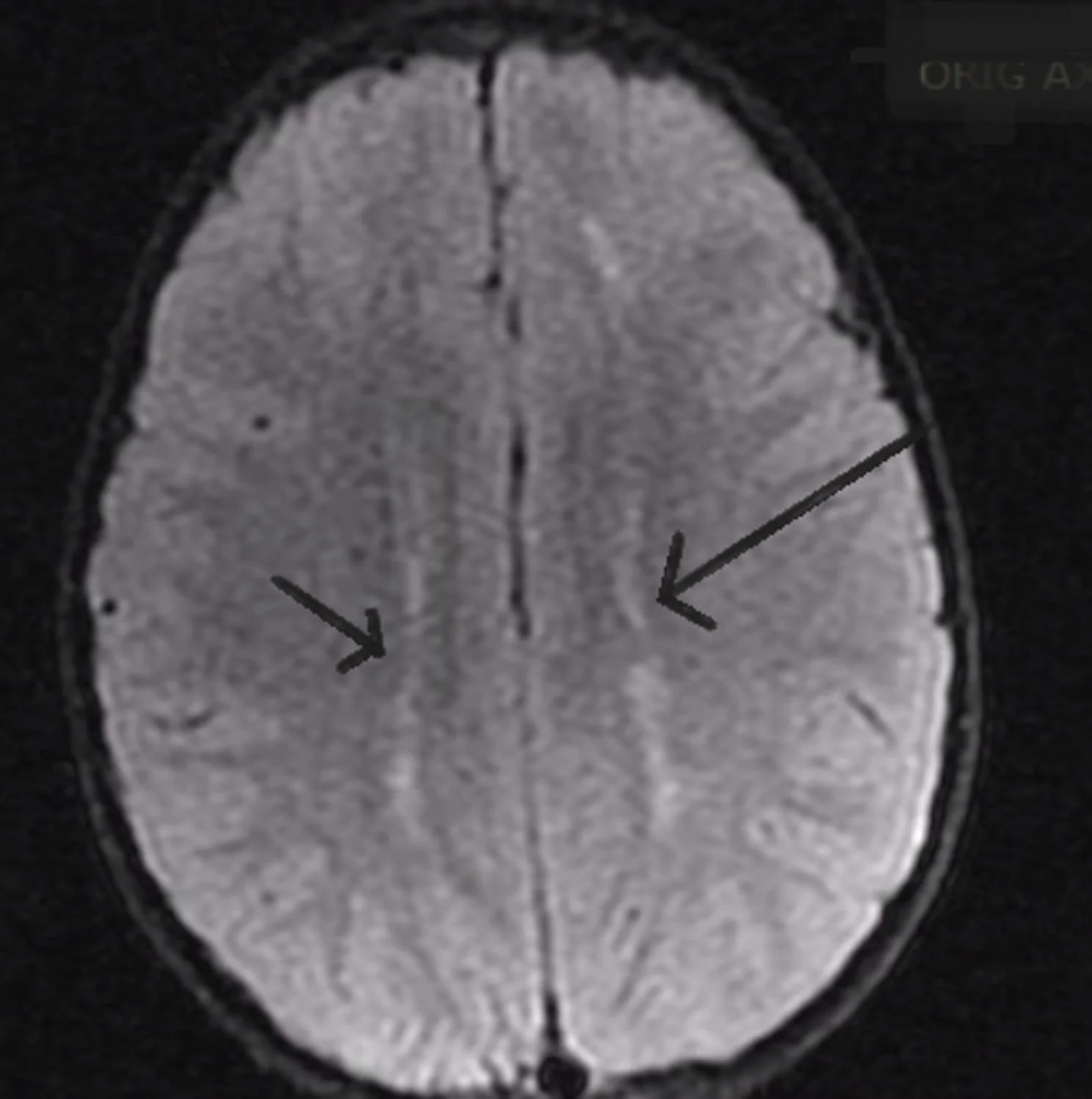
Axial T2-weighted brain MRI shows linear hyperintensity in the white matter within the bilateral watershed region (Arrows)

Vital signs were normal, with a blood pressure of 102/53 mmHg. Blood glucose and serum electrolytes were within normal limits. Brain Computed Tomography (CT) scan was unremarkable. Electroencephalography (EEG) showed background slowing without epileptiform activity. Cerebral spinal fluid (CSF) analysis was normal. Contrast-enhanced Magnetic Resonance Imaging (MRI) was performed after 24 h of admission, and demonstrated bilateral watershed infarctions, approximately symmetric, with greater involvement of the left cerebral hemisphere, located between the anterior and middle cerebral artery territories and to a lesser extent between the middle and posterior cerebral artery territories. The initial diagnostic MRI protocol consisted of T2-weighted and fluid-attenuated inversion recovery (FLAIR) sequences. Diffusion-weighted imaging (DWI) and apparent diffusion coefficient (ADC) sequences were not included in the initial MRI protocol at the imaging center. The infarcts involved only the white matter and did not involve the cerebral cortex (Fig. 1), and Magnetic Resonance Angiography (MRA) revealed no vascular occlusion.

**Fig. 2:**
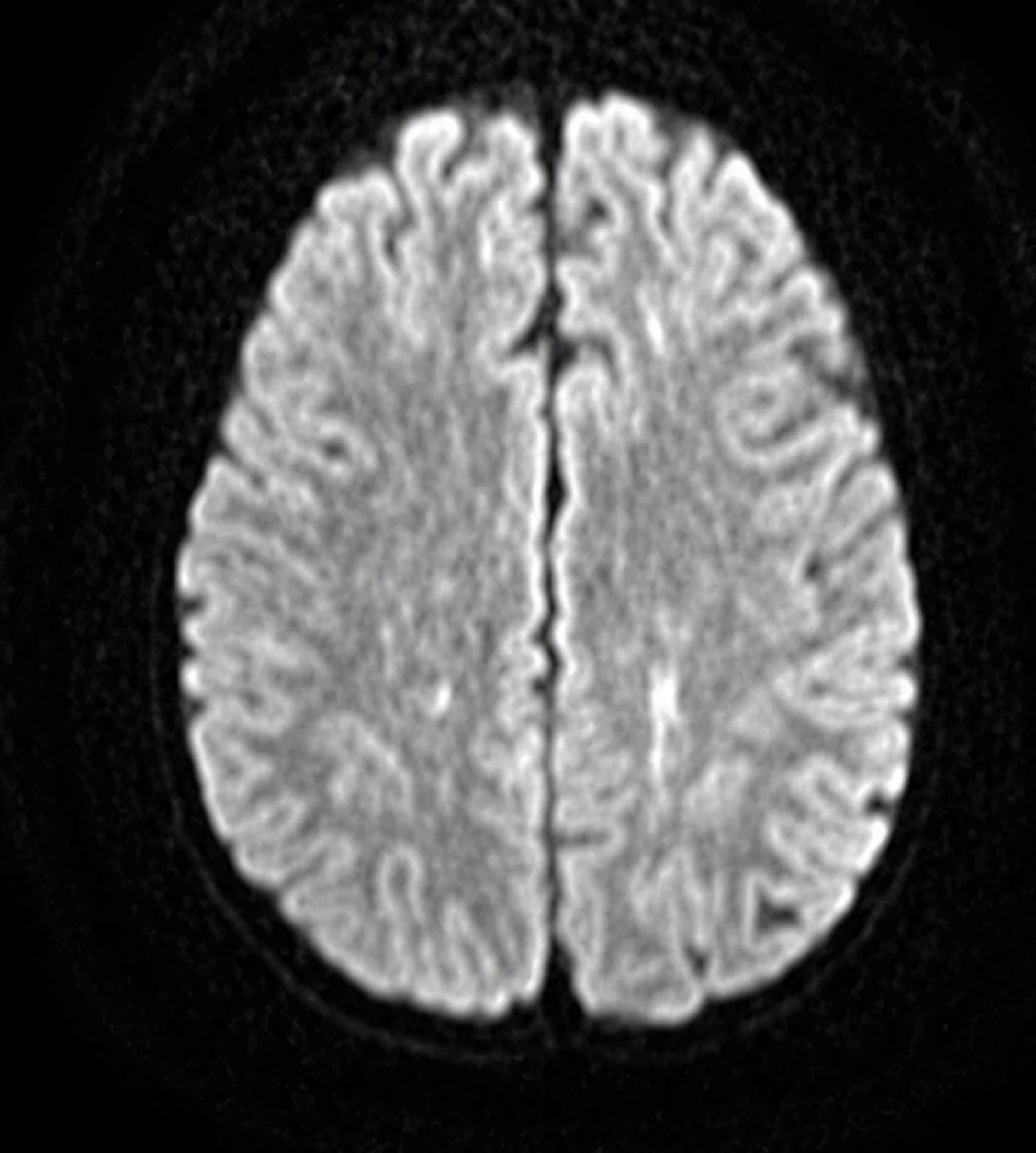
Axial DWI image showing a focal hyperintense lesion in the left posterior parietal region, compatible with diffusion restriction

With intensive supportive care, including hemodynamic monitoring, maintaining hemoglobin levels above 10 g/dL, and keeping electrolyte values within the normal range, neurological improvement began within 72 h. The patient regained full consciousness (GCS 15), with motor strength improved to a full 5/5 in the upper limbs and 4/5 in the lower limbs, and ambulation with a wide-based gait. Speech became clear and complete neurological recovery occurred within eight days, confirmed by normal examination and follow-up MRI which was performed at day-6 of admission, showing an improvement in the previous ischemic findings, where the previously observed hyperintense focus became less conspicuous with poorly defined borders. The MRI sequences included in the second examination were T2, FLAIR, DWI and ADC, with the infarcted regions appearing as hyperintense areas on DWI with corresponding diffusion restriction (Fig. 2). The patient was discharged in good condition with complete recovery at day-9 of admission, after being provided with a list of medications and foods to avoid, along with a recommendation for genetic counseling of family members to facilitate the identification of subclinical cases. After two months of follow-up, with adherence to the G6PD deficiency diet, no complications, neurological symptoms, or new episodes of hemolysis were observed. The diagnosis was confirmed by assaying G6PD in the blood 3 months after the hemolytic crisis, which measured 0.51 U/g Hb (normal range 5-20.5). The latest complete blood count showed significant hematologic improvement, with a hemoglobin level of 11.4 g/dL, a white blood cell count of 8,490/mm^3^, 25% neutrophils, and a platelet count of 353 × 10³/mm^3^.

## Discussion

Watershed infarction resulting from hemolytic anemia was first reported by Enzmann et al. in 1976 [2]. Since then, it has been frequently reported in the context of hemolytic disorders [1, 7]. However, none of these cases was attributed to favism. Ischemic stroke has b een studied and reported in several studies in association with hematologic diseases [3, 4, 8]. Moreover, none of them was attributed to favism. There is limited information in the literature regarding the association between G6PD deficiency and cerebral infarction. A literature search was conducted in PubMed and Google Scholar without date restrictions. In PubMed, Medical Subject Headings (MeSH) and free-text keywords were used. The search strategy included the terms “Glucosephosphate Dehydrogenase Deficiency” [MeSH], “Anemia, Hemolytic” [MeSH], and related keywords (“G6PD deficiency,” “glucose-6-phosphate dehydrogenase deficiency,” “favism,” and “hemolytic anemia”), combined with (“crisis” OR “attack”) and (“watershed infarction” OR “ischemic stroke”). No restrictions were applied regarding language, patient age, publication date, or article type, and all publication types were considered. Only a few studies have investigated how the coexistence of G6PD deficiency and sickle cell anemia affects the prevalence and clinical outcomes of stroke [8], however, their results were contradictory. Two studies have suggested an association between G6PD deficiency and unfavorable outcomes in stroke patients [4, 9]. These findings were observed through screening for G6PD deficiency in stroke patients, rather than during ischemic stroke episodes triggered by favism. In this article, we report an extremely rare case of watershed infarction reported after favism crisis.

Some authors have studied the outcomes and risk factors of stroke in adults with hemolytic anemia [1, 4, 9]. Other studies have demonstrated this relationship between stroke and hemolytic anemia in children [2, 3, 8, 9]. Furthermore, hemolytic crises secondary to favism have demonstrated greater clinical severity in pediatric patients, and this was potentially attributable to their lower body mass [6]. However, we present a case of a 3-year-old boy recovered from a sever favism crisis with no remarkable complications following appropriate treatment. The symptoms of watershed infarction appeared at hemoglobin level of 3.4 g/dL in a 25-year-old female [1], while neurological symptoms without any evidence of infarction or stroke appeared at hemoglobin levels 3 and 8.4 g/dL [6]. The most common cause of watershed infarction in hemolytic anemia patients is hypotension [1, 2], although it occurs in cases of severe hemolytic crises due to severe anemia without hypotension [1], which coexisted with chronic intracranial carotid occlusion proved by MRA. However, we appreciate that the acute reduction in hymoglobin was the cause of the watershed infarction that occurred in our patient because the hemodynamics were within the normal limits at the time of symptom onset, despite transient hypotension and hypoxia at the time of admission which automatically improved after fluid resuscitation and blood transfusion.

In the pathophysiological context, multiple factors contribute to the pathogenesis of stroke in sickle cell disease, including instability of vascular tone, susceptibility to fluctuations in nitric oxide levels, and diminished cerebrovascular reserve capacity. Additionally, endothelial injury—likely resulting from abnormal erythrocytes—along with a generalized elastic tissue defect and fragmentation of the internal elastic lamina of the arterial walls, has also been reported [3]. Prior research has suggested that G6PD deficiency may contribute to direct vascular injury. Reduced G6PD expression has been associated with increased accumulation of reactive oxygen species (ROS) and oxidative injury in endothelial cells, highlighting the essential role of G6PD in regulating the vascular redox state. Furthermore, G6PD deficiency has been shown to diminish endothelial nitric oxide bioavailability, thereby contributing to vascular dysfunction, which may in turn play a role in the development of cerebral ischemia [4]. While the pathologies in beta-thalassemia include a hypercoagulable state, cardioembolism, and large-vessel thrombosis [1]. In the present case, the combined contribution of multiple factors, including mild hypotension, transient hypoxia, and severe acute anemia, may represent the most likely pathogenic mechanism. However, no conclusive evidence currently supports this hypothesis.

The diagnosis of G6PD deficiency is dependent on clinical presentations, laboratory findings, and family history [5, 6]. Definitive diagnosis is performed through genetic testing [5, 8], or by measuring G6PD level, a method frequently employed in clinical practice [4, 6, 8]. MRI is the diagnostic test of choice for watershed infarction [1, 7, 9], with hyper signal intensity [1] on Flair and T2-weighted MRI [7, 10]. MRA showed chronic occlusion of the left internal carotid artery in one reported case [1]. EEG was conducted in one case and showed diffuse slow waves [2]. The same author performed a lumbar puncture, and it was unremarkable. In this article, the diagnosis was based on clinical presentations and family history, and was confirmed by measuring G6PD blood level 3 months after the hemolytic crisis which found to be 0.5 U/g Hb. However, genetic and DNA testing are unavailable in our country. CT scan, lumbar puncture and MRA were performed in our case at the onset of neurological symptoms and were normal, EEG showed mild background slowing, and the diagnosis of watershed infarction was made by MRI which revealed regions of hyperintensity in the left and right watershed area on T2, Flair (Fig. 1). Although a cardiac murmur was detected during the initial physical examination, echocardiography was not performed to exclude underlying structural cardiac abnormalities. A chest radiograph demonstrated no abnormalities in cardiac size or silhouette. In the absence of radiographic evidence of cardiac pathology, the murmur was presumed to be secondary to severe acute anemia. With regard to the laboratory evaluation for thrombotic disorders, platelet count, PT, and PTT were assessed and found to be within normal limits. Further thrombophilia testing was not performed because there was no clinical suspicion of an underlying thrombotic disorder. Cerebral stroke in hemolytic anemia tends to be asymptomatic [3], but it may present with neurological symptoms such as hemiparesis, headache, seizures [1, 2], aphasia and coma [2]. The most optimal treatment of stroke in hemolytic patients is blood transfusion [1] and fluid resuscitation [11]. With the highlight of the importance of accurate blood amounts, particularly in children, to avoid repeated blood transfusions [11]. In the presented case, the patient has undergone two episodes of packed RBCs of 15 mL/kg per dose. The differential diagnosis of pediatric stroke includes migraine, functional neurological disorders (previously referred to as psychogenic disorders), musculoskeletal abnormalities, intracranial infection, inflammatory disorders of the central nervous system, and intracranial tumors. In most cases, these conditions can be distinguished using computed tomography (CT) and magnetic resonance imaging (MRI), with additional laboratory investigations guided by the clinical presentation. MRA imaging may be useful to confirm arterial occlusion or identify underlying cerebrovascular abnormalities [12]. Most patients who have undergone appropriate treatment and follow-up revealed no complications during different periods of follow-up [1, 5]. The follow-up period for stroke due to hemolytic disorders ranged from 2 weeks [2] to several years [3], some of these follow-up periods revealed complications, including cortical blindness [2] and, in some cases, death [3], while others did not report any complications. However, our patient was discharged after full recovery and followed up for three months without any complications or neurological dysfunctions.

## Conclusion

To the best of our knowledge, this is reported case of watershed infarction associated wit favism. We emphasize the importance of accurate and frequent neurological examination of patients experiencing hemolytic crises until discharge, as well as the need for appropriate and early treatment to prevent significant adverse complications.

## Supplementary Information

Supplementary Material 1.

## Data Availability

All data of the patient generated during this study are included in this published article and its supplementary information files.

## Abbreviations

G6PD: Glucose-6-phosphate dehydrogenase
RBCs: Red Blood Cells
PT: Prothrombin Time
PTT: Partial Thromboplastin Time
GCS: Glasgow Coma Score
CT: Computed Tomography
EEG: Electroencephalography
CSF: Cerebral Spinal Fluid
DWI: Diffusion-Weighted Imaging
ADC: Apparent Diffusion Coefficient
MRI: Magnetic Resonance Imaging
FLAIR: Fluid-attenuated inversion recovery
MRA: Magnetic Resonance Angiography
ROS: Reactive Oxygen Species

## References

[1] Ansari B, Saadatnia M, Okhovat AA. Watershed infarct in beta-thalassemia major patient. Case Rep Neurol Med. 2017;2017:2736402. 10.1155/2017/2736402 PMC5385913 28428899

[2] Enzmann D, Gates GF. Watershed infarction in sickle cell disease. Radiology. 1976;118(2):337–9. 10.1148/118.2.337 766056

[3] Salih MA, Abdel-Gader AM, Al-Jarallah AA, Kentab AY, Alorainy IA, Hassan HH, Khoja WA. Hematologic risk factors for stroke in Saudi children. Saudi Med J. 2006;27(Suppl 1):S21–34. 16532128

[4] Ou Z, Chen Y, Li J, Ouyang F, Liu G, Tan S, Zeng J. Glucose-6-phosphate dehydrogenase deficiency and stroke outcomes. Neurology. 2020;95(11):e1471–8. 10.1212/WNL.0000000000010245 32651291

[5] Sköld MB, Svendsen RP, Pedersen EB. Favism after ingestion of fava beans in a three-year-old child with glucose-6-phosphate dehydrogenase deficiency. Ugeskr Laeger. 2017;179(20):V01170004. 28504635

[6] Beretta A, Manuelli M, Cena H. Favism: clinical features at different ages. Nutrients. 2023;15(2):343. 10.3390/nu15020343 PMC9864644 36678214

[7] Stotesbury H, Hales PW, Hood AM, Koelbel M, Kawadler JM, Saunders DE, et al. Individual watershed areas in sickle cell anemia: an arterial spin labeling study. Front Physiol. 2022;13:865391. 10.3389/fphys.2022.865391 PMC9110791 35592036

[8] Belisário AR, Rodrigues Sales R, Toledo NE, Velloso-Rodrigues C, Silva CM, Viana MB. Glucose‐6‐Phosphate Dehydrogenase deficiency in Brazilian children with sickle cell anemia is not associated with clinical ischemic stroke or high‐risk transcranial Doppler. Pediatr Blood Cancer. 2016;63(6):1046–9. 10.1002/pbc.25924 26840990

[9] Li J, Chen Y, Ou Z, Zhang Y, Liang Z, Deng W, Zeng J. Glucose-6-phosphate dehydrogenase deficiency and intracranial atherosclerotic stenosis in stroke patients. Eur J Neurol. 2022;29(9):2683–9. 10.1111/ene.15418 35608963

[10] Wright JN, Shaw DWW, Ishak G, Doherty D, Perez F. Cerebellar watershed injury in children. AJNR Am J Neuroradiol. 2020;41(5):923–8. 10.3174/ajnr.A6532 PMC7228150 32327437

[11] Tarhani F, Nezami A, Heidari G, Abdolkarimi B. Clinical manifestations and therapeutic findings of the children with glucose-6-phosphate dehydrogenase deficiency presenting favism. Endocr Metab Immune Disord Drug Targets. 2021;21(6):1125–9. 10.2174/1871530320999200818182905 32811422

[12] Marcdante KJ, Kliegman RM, editors. Nelson Essentials of Pediatrics. 8th ed. Philadelphia: Elsevier; 2019.

