# Supplementary material for: Watershed infarction during the first favism crisis in a Syrian child; a rare case report
Source: BMC Pediatr. 2026 Aug 19;26:1176. doi: 10.1186/s12887-026-07550-7 (PMC13761087; doi:10.1186/s12887-026-07550-7)
Supplement: Supplementary file 1 [file 12887_2026_7550_MOESM1_ESM.docx]

The patient

بش

Male

Female

Affected
